# Reconciling actual and perceived rates of predation by domestic cats

**DOI:** 10.1002/ece3.1553

**Published:** 2015-06-19

**Authors:** Jennifer L McDonald, Mairead Maclean, Matthew R Evans, Dave J Hodgson

**Affiliations:** 1 Centre for Ecology and Conservation, College of Life and Environmental Sciences, University of ExeterPenryn, Cornwall, TR10 9FE, UK; 2 School of Biological and Chemical Sciences, Queen Mary University of LondonMile End Road, London, E1 4NS, UK

**Keywords:** Cat regulation, domestic cats, owner perception, predation, stakeholder conflict, wildlife conservation

## Abstract

The predation of wildlife by domestic cats (*Felis catus*) is a complex problem: Cats are popular companion animals in modern society but are also acknowledged predators of birds, herpetofauna, invertebrates, and small mammals. A comprehensive understanding of this conservation issue demands an understanding of both the ecological consequence of owning a domestic cat and the attitudes of cat owners. Here, we determine whether cat owners are aware of the predatory behavior of their cats, using data collected from 86 cats in two UK villages. We examine whether the amount of prey their cat returns influences the attitudes of 45 cat owners toward the broader issue of domestic cat predation. We also contribute to the wider understanding of physiological, spatial, and behavioral drivers of prey returns among cats. We find an association between actual prey returns and owner predictions at the coarse scale of predatory/nonpredatory behavior, but no correlation between the observed and predicted prey-return rates among predatory cats. Cat owners generally disagreed with the statement that cats are harmful to wildlife, and disfavored all mitigation options apart from neutering. These attitudes were uncorrelated with the predatory behavior of their cats. Cat owners failed to perceive the magnitude of their cats’ impacts on wildlife and were not influenced by ecological information. Management options for the mitigation of cat predation appear unlikely to work if they focus on “predation awareness” campaigns or restrictions of cat freedom.

## Introduction

The threat posed by domestic cat (*Felis catus*) predation to native biodiversity is gaining increasing recognition (Woods et al. 2003; Baker et al. 2008; Loss et al. 2013; Loyd et al. 2013), together with the realization that developing mitigation measures requires cooperation from cat owners (Lilith et al. 2006; van Heezik 2010; Thomas et al. 2012). Globally, cats are responsible for killing a range of native wildlife (Bonnaud et al. 2011) including herpetofauna (Arnaud et al. 1993), invertebrates (Medina and García 2007), birds (Blancher 2013), and small mammals (Woods et al. 2003), many of which are endangered. In contrast to natural predators, domestic cats are not reliant on prey availability to meet their daily energy demands and can attain densities far higher than the natural carrying capacity of their environment because their owners provide them with food (Beckerman et al. 2007). This, combined with their impulsive predatory instinct, poses a sizeable threat to prey populations (May 1988; Woods et al. 2003; Baker et al. 2008; van Heezik et al. 2010; Loss et al. 2013). Estimates of the number of animals killed every year by domestic cats are in the magnitude of millions in the UK (Woods et al. 2003) and Canada (Blancher 2013), and billions in the United States (Loss et al. 2013). Quantifying the ecological consequences of domestic cat ownership has been the focus of many studies, but our understanding of the perceptions and attitudes of cat owners has lagged behind. In the UK alone, there are over 10 million domestic cats residing in 23% of households (Murray et al. 2010; Thomas et al. 2012). The opposing roles of cats, as both human companions and wildlife predators, are likely to drive divergent interests between cat owners and conservationists and may develop into a socially intractable problem should mitigation strategies be required.

In the USA, management largely focuses on controlling feral cat populations, although conservation groups also run initiatives such as the American Bird Conservancy’s “Cats Indoors Campaign” (Dauphiné and Cooper 2009). In Australia, engagement with cat owners at both state and local government levels is more apparent, and highlighting welfare advantages (Grayson and Calver 2004; Lilith et al. 2006) has enabled stricter management to be enforced locally, including cat containment and cat curfews near nature reserves (Denny and Dickman 2010). Enforcing mitigation measures is predominantly a social issue complicated by discordant attitudes among stakeholders (Farnworth et al. 2014), for example, cat colony caretakers and bird conservationists have polarized views regarding the impacts of feral cats on wildlife (Peterson et al. 2012). The conflicting roles of conservationists, concerned with native wildlife populations, and cat owners, concerned about the implications for their pets, are significant obstacles to overcome for effective management. Owning a cat unsurprisingly alters individual attitudes toward proposed management strategies (Grayson et al. 2002; Thomas et al. 2012), and owners are unlikely to favor management that restricts cat freedoms or is detrimental to cat welfare. Addressing these attitudes will be an essential step to help plan conservation measures should they ever be deemed necessary within the UK (van Heezik 2010). It remains unclear whether incentives for management strategies should be communicated in the form of ecological evidence or in terms of cat welfare. Focussing on gaps in current knowledge, we survey the perceptions of cat owners regarding the predatory behavior of their cats and also consider their opinions on the wider issue and acceptability of control measures.

Although cat personality is responsible for a large amount of variation in cat hunting behavior and prey specialization (Dickman and Newsome 2014), studies monitoring the predation rates of domestic cats (Baker et al. 2008; Thomas et al. 2014) have generally shown that cats in better states of physical fitness, that is, younger (Churcher and Lawton 1987; Woods et al. 2003; van Heezik et al. 2010) and leaner (Woods et al. 2003), catch and return more prey. Additionally, localized habitat differences have been shown to drive variability in prey encounters, that is, hunting rates largely reflect immediate prey availability (van Heezik et al. 2010; Loyd et al. 2013) and proximity to potential prey sources (Barratt 1998). In the UK, farmland habitat may act as a proxy for prey availability with field boundaries providing foraging and nesting sites (Peach et al. 2004), and hence promoting species diversity and wildlife abundance (Baker and Harris 2007). Evaluating the impact (or lack thereof) of proximity to farmland on predation rates may provide further support to the body of evidence, suggesting that local landscape heterogeneity drives predation rates. In addition, identifying traits that increase predation risk could be used to advise owners of the “predatory” potential of their cat and prioritize conservation efforts in locations where wildlife is under particular threat.

Understanding the predatory behavior of domestic cats is clearly important, with results from such studies having the potential to be used as an advisory tool to aid targeted management. There is also, however, a clear need to directly address the perceptions and opinions of cat owners. We consider owners’ views regarding their cats’ predatory behavior by asking whether owners’ predictions of the number of prey their cat returns correlate with actual numbers bought home. Additionally, we assess whether the predatory behavior of cats influences the attitudes of their owners on the wider ecological consequence of domestic cat ownership and proposed control strategies. A naive hypothesis would be that owners of highly predatory cats are more likely to agree that cats are harmful to wildlife. However, any attachment between owner and cat might defy any decision-making based upon ecological rationale. Addressing both the predatory behavior of cats and their owners’ perceptions, we aim to test this hypothesis and provide a better understanding of the ecological and societal issue of domestic cat ownership.

## Methods

Mawnan Smith, England, is the principle site of this study. Here, data were collected on the accuracy of owners at predicting their cats’ prey-return rates, their attitudes toward the wider issue of domestic cat predation, and an investigation into the drivers of predation rates in domestic cats. In addition to this, a separate study was implemented at Thornhill, Scotland. As part of this study, cat owners were asked to predict the amount of prey their cat would return, providing the opportunity to review the accuracy of owners at predicting their cats’ prey-return rates across two separate study sites.

### Study areas and participant predictions

#### Mawnan Smith

Mawnan Smith, Cornwall, England, a village of approximately 3.8 square kilometers was chosen as a study site to represent a predominantly isolated rural community surrounded by farmland habitat.

Cat owners were recruited to the study by delivering participation forms to residences throughout the village. Responses were received from 31 households (7.9% of estimated total households) totaling 43 cats that all took part in the prey-returned study. Prior to survey commencement, owners were also asked to predict the number of prey their cat would return per month based on the surveying period. In cases where volunteers did not follow the questionnaire instructions, the data were edited as follows: If a numeric range was given, the average was used, and if respondents left an answer blank, then their data were omitted from the respective analysis.

Prey items returned home were recorded by owners over a 4-month period (1st March–30th June 2010). Prey-recording forms were collected and distributed on a monthly basis to maintain participant motivation throughout the study.

#### Thornhill

Thornhill is a small rural village, surrounded by pastoral farmland, situated approximately 15 miles northwest of the city of Stirling, Scotland. A door-to-door survey of all houses was undertaken to inform residents of the study. A total of 27 households responded (13.5% of estimated total households), consisting of 43 cats, which all took part in the study. At the start of the study, each owner was asked to estimate the number of prey their cat caught per year based on the numbers returned previously. Owners typically expressed the number of prey their cat killed in terms of fixed durations, that is, total prey returned per week, month, or year. Predictions were subsequently standardized to provide a monthly estimate. Owners recorded prey returns over a 14-month period (July 2003 – August 2004) and were visited on a monthly basis with replacement data sheets provided when necessary. When it was not possible for cat owners to collect data continuously throughout the year, we calculated a monthly prey-return rate from the data available for each cat.

The following data collection and corresponding analyses focus on information collected from the Mawnan Smith study area.

### Owner perception survey

We designed the survey to determine cat owner attitudes toward the ecological impact of domestic cats, to propose control strategies, and to identify the influence of the predatory behavior of their own cat(s) on their responses. Each of the 31 households taking part in the study was surveyed, and multiple surveys were provided to households with more than one occupant. The survey was administered to 45 cat owners following completion of the prey-return survey to get their responses in the context of the actual predatory behavior of their cat. The survey used a four-point Likert scale based on questions used in previous studies (Lilith et al. 2006).

1. Domestic cats killing wildlife is a serious problem
2. All cats should be neutered
3. Domestic cats are harmful to wildlife
4. I would be happy to keep my cat(s) on my property between sunset and sunrise
5. I would be happy to keep my cat(s) on my property at all times

The survey was analyzed by assigning scores 1–4 to responses to each question (1 = strongly agree, 2 = agree, 3 = disagree, and 4 = strongly disagree).

### Study of drivers of predatory behavior

To determine key influences of prey-return rates, ancillary information about the cats was compiled including age, sex, estimated time spent outside (hours), presence of a bell (Y/N), food type (wet and/or dry), and whether they were allowed outside at night (Y/N). Distance from farmland was also calculated, using GPS coordinates at each cat’s residence and at the nearest farmland border (edge of village) (see Appendix (Table3) for further details of predictor variables).

### Statistical analysis

For both study areas, a chi-square test was initially used to see if owners were aware whether their cat would return prey or not (as indicated by a predicted prey return >0). Spearman’s rank correlations were then used to test the correlation between monthly predation rates obtained for each cat and the number of prey that owners estimated would be returned by their cat. Focussing on the Mawnan Smith area, the owner perception survey was summarized using standard descriptive statistics. To analyze the variation in Likert scores, we used generalized linear mixed models (GLMM) with residence as a random effect, due to more than one respondent for some households, and a binary error structure, to account for owner willingness to agree (0) or disagree (1) with survey questions, with the average prey-return rates of their cat(s) as predictor variables.

The impact of covariates on prey returns was analyzed using generalized linear mixed modeling (GLMM), accounting for multiple cat households as a random effect, and using a Poisson’s error structure, to represent counts of prey items, using the package lme4 (Bates et al. 2013) in R version 3.1.1 (R Development Core Team, 2014). Where several cats lived in the same house, it was not always possible to assign prey returned to a particular cat. The statistical analysis included only those prey returns that could be assigned to individual cats. We used the function “dredge” of the package “MuMIn” (Barton 2013) to compare all variations of models containing the explanatory variables age, sex, “distance to farmland”, food type, “presence of a bell”, “kept inside at night,” and “time spent outside”. The candidate model set consisted of all possible combinations of these explanatory variables. Following Burnham and Anderson (2002), candidate models were assessed using AICc to account for the small sample size (*n*) relative to the number of parameters (*k*), wherein *n*/*k* < 40. We interpreted the influence of each variable using its cumulative AICc weight in addition to the model averaged effect size (and associated confidence intervals). Cumulative AICc weights represent the proportion of weight attributable to models containing that particular variable and are calculated by summing the AICc model weights of all models containing that variable. Continuous covariates were standardized to have a mean of 0 and standard deviation of 1 to emphasize the relative strength of the regression coefficients.

## Results

### Prey returns and owners’ predictions

**Figure 1:**
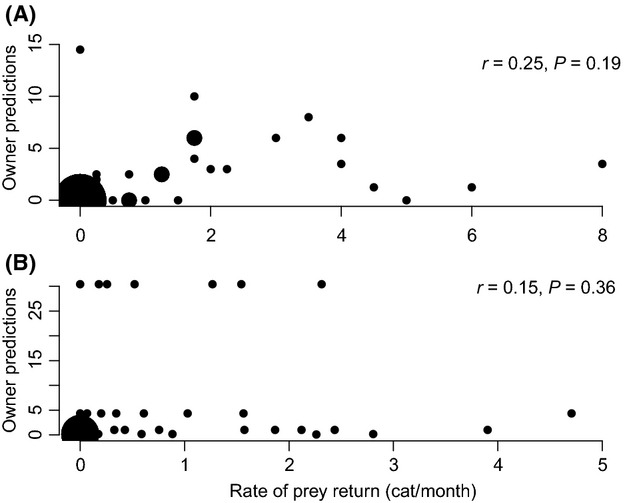
Owners predictions and average monthly prey returns at (A) Mawnan Smith and (B) Thornhill (rates of prey return are not shown when owners failed to provide a prediction) along with the degree of correlation (see inset correlation coefficient *r* and corresponding *P*-value). Size of point is proportional to the number of overlapping data.

Of the 43 cats surveyed at the Mawnan Smith site, 10 did not return any prey during the 4-month study period. The majority of owners accurately predicted whether their cat would return prey or not (contingency test, 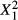 = 7.2, *P* < 0.01), but there was no correlation between observed and perceived prey returns of predatory cats (Spearman’s *r* = 0.25, *P* = 0.19, Fig.1A). Average prey returned per cat per month ranged from 0 to 10.25 (mean 1.89 ± 0.35 SE, Fig.1). Over the 4-month period, 325 prey items were returned. Mammals were the most common prey captured (58.6%). Rodents (mice, voles and rats) accounted for the majority (57.3%) of all mammalian prey, followed by shrews (30.8%). Birds accounted for 26.5% of all records, with the house sparrow (*Passer domesticus*) the most common bird species contributing to 18% of all birds returned; reptiles contributed 8.7% of prey recordings. The remaining 6.2% of prey returned were unidentifiable.

**Figure 2:**
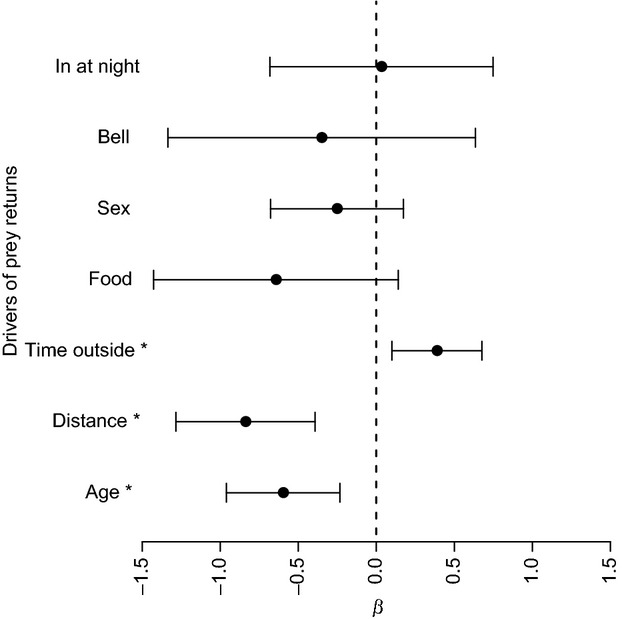
The effect size (distance from zero) and direction of drivers of prey returns along with their 95% confidence intervals predicted from a GLMM with model averaged beta-parameters. Predictors that were identifiably different from zero are indicated by *.

At the Thornhill site, 15 of 43 cats did not return any prey. Similar to the Mawnan Smith site, cat owners were accurate at determining whether their cat would return prey or not (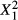 = 6.02, *P* = 0.04), but their estimates did not correlate with actual monthly prey returns of predatory cats (Spearman’s *r* = 0.15, *P* = 0.36, Fig.1B). Average prey returned per month ranged from 0 to 4.75 prey items (Mean 0.81 ± 0.17 SE, Fig.2). Mammals were the most common prey returned (72.8%). Mammalian prey consisted mainly of rodents (75.8%) and shrews (17.7%). Birds accounted for 26.3%, with the house sparrow contributing 34% of all bird recordings, and a frog (0.3%), with the remaining prey items (0.6%) unidentifiable.

Native species made up the majority of prey returned by cats at both studies. Only 15.7% and 6.1% of species returned at Mawnan Smith and Thornhill, respectively, were identified as non-native, these included the brown rat (*Rattus norvegicus*) and European rabbit (*Oryctolagus cuniculus*).

### Perceptions of cat owners

The predatory behavior of cats did not influence whether their owners agreed or disagreed with cat containment at night (GLMM: 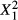 = 0.97, *P* = 0.32) or at all times (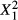 = 0.13, *P* = 0.72). Additionally, their views on whether domestic cats are harmful or a serious problem to wildlife was not related to the predatory behavior of their cat (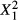 = 0.36, *P* = 0.54; 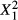 = 0.13, *P* = 0.73). Cat owners largely disagreed with all statements, with the exception of sterilisation, with 62% agreeing or strongly agreeing that all cats should be neutered (Table1).

**Table 1:** Percentage distribution of owners’ responses to domestic cat predation and control in order of agreement from most (1) to least agreeable (5). Note that the majority of respondents disagreed with statements 2–5

Question	Response			
	Strongly disagree	Disagree	Agree	Strongly agree
1. All cats should be sterilised	11	27	44	18
2. Domestic cats are harmful to wildlife	13	47	33	7
3. I would be happy to keep my cat on my property between sunset/sunrise	20	41	30	9
4. Domestic cats killing wildlife is a serious problem	20	53	22	5
5. I would be happy to keep my cat on my property at all times	46	52	2	0

Five owners felt the need to add unsolicited responses on the questionnaires themselves, further highlighting their negative perceptions regarding cat control. Owners are strongly opposed to keeping their cats in at all times: 98% of owners disagreed with this control strategy, of which 46% strongly disagreed, and the following comments were provided “You can’t keep cats in at all times” and “My cat chooses for herself whether to stay in or go out”. The majority (60%) of cat owners disagreed that cats are harming wildlife, of which 13% strongly disagreed, and additional opinions were provided, including “but it’s nature”, “some wildlife is harmful to cats,” and “but other wildlife is harmful to wildlife”.

### Drivers of prey returns

Younger cats, those residing closer to farmland and those who were estimated to spend more time outside, were likely to return more prey (Fig.2; Table2). These drivers were observed to be most important, as indicated by model averaged beta-parameters (Fig.2) and the highest cumulative AICc weights (*c*): estimated time spent outside (*c* = 0.998), distance to farmland (*c* = 0.991), and age (*c* = 0.985). The variables less influential, with 95% confidence intervals of model averaged beta-values spanning zero (Fig.2) and lower cumulative AICc weights, were presence of a bell (*c* = 0.289), food type (*c* = 0.464), sex (*c* = 0.315), and whether the cat was kept inside at night (*c* = 0.186).

**Table 2:** Candidate model set of generalized linear mixed models with delta-AIC < 4 (Burnham and Anderson 2002) exploring the effect of covariates on prey-return rates, including the cats’ age, sex, distance of residence from farmland, estimated time spent outside per day, food choice (wet and/or dry), presence of a bell, and whether they are kept in at night

Model	df	logLik	AICc	Δ AIC	AIC weight
Age + Distance + Outside	5	−110.587	232.9	0	0.253
Age + Distance + Outside + Food	6	−109.304	233.2	0.22	0.227
Age + Distance + Outside + Sex	6	−109.957	234.5	1.52	0.118
Age + Distance + Outside + Food + Sex	7	−108.604	234.7	1.77	0.104
Age + Distance + Outside + Bell	6	−110.378	235.3	2.36	0.078
Age + Distance + Outside + Bell + Food	7	−109.066	235.6	2.69	0.066
Age + Distance + Outside + Inside Night	6	−110.579	235.7	2.76	0.064
Age + Distance + Outside + Inside Night + Food	7	−109.303	236.1	3.17	0.052
Age + Distance + Outside + Bell + Sex	7	−109.628	236.8	3.82	0.038

## Discussion

Domestic cat predation is a divisive issue driven by the different motivations of cat owners and conservation biologists. We have illustrated how owners fail to perceive the ecological footprint of their cat, and have shown that their opinions on the general problem are not influenced by the predatory behavior of their cat. We have demonstrated that cat owners in this study reject the proposition that cats are a threat to wildlife, and oppose management strategies with the exception of neutering. These results can be taken forward to build a fuller understanding of owners’ perspectives and ultimately develop collaborative mitigation measures.

Cat owners were broadly aware whether their cat was predatory or not, but they were unable to perceive the magnitude of predation among predatory cats, with a dissociation between actual and perceived predatory behavior found across both study areas. A mere ownership effect may be responsible for the distorted assessment of their cats’ predatory habits, whereby overly favorable views of owned possessions are extended to pets (El-Alayli et al. 2006). This could result in overestimation or underestimation of their cats’ predatory prowess, depending on each owner’s appraisal of what constitutes a favorable trait.

Although these data suggest conservationists should address the perceptions of cat owners, owners dissociated themselves from conservation responsibilities with attitudes independent of the ecological impact of their cat. Therefore, based on the opinions of cat owners in this study, challenging the perceptions of owners regarding their own cats’ predatory behavior is unlikely to influence their point of view regarding cat predation and control initiatives. The majority of cat owners disagreed that cats were a problem or harmful to wildlife, and were against proposals of containment as a control measure. It is perhaps unsurprising that owners provided negative responses especially as there is little evidence that cats are affecting prey populations [but see Bamford and Calver (2012) and Dufty (1994)]. Furthermore, the majority of cats only return a small amount of prey; instead, it is the cumulative effect of high densities of cats that have an overall negative effect on the environment [not only in direct predation, but also due to indirect sublethal effects (Beckerman et al. 2007)]. Therefore, along with an appreciation of their cat’s predatory behavior, cat owners may also need to apprehend how individual predation rates scale up with increased cat densities to perceive the negative impact of cats on wildlife. These results, along with further unsolicited responses, emphasize the strong differences in objectives between conservationists and cat owners and further suggest that some cat owners may have distorted views regarding their cats’ place in the environment: Comments implied that cat predation is a natural ecosystem interaction.

Although we cannot decisively conclude whether the opinions that we observed accurately represent nonrespondents (i.e. participant bias), or represent attitudes at a national level, our results are comparable with those from a separate UK study (Thomas et al. 2012). The majority of cat owners (68%) surveyed in an urban area considered cats to have either no or a small influence on bird populations (Thomas et al. 2012), a figure comparable to the disagreement of rural residents that cats were harmful to wildlife (60%) and that cats killing wildlife is a serious problem (73%). Similarly, equal levels of acceptability for compulsory sterilisation were found across both studies (61% (Thomas et al. 2012), compared to our finding of 62% in a rural area). In both our study, and that of Thomas et al. (2012), the assessment of attitudes of cat owners was one part of a wider study. Further studies that more explicitly address the attitudes of cat owners using multiple statements per question and addressing a greater range of management strategies over a larger geographic would be beneficial.

In common with previous studies, we assumed prey returns to be associated with actual prey kills. However, cats are thought to kill up to three times more prey then they bring back, either because they consume or abandon their kills at the capture site (Kays and Dewan 2004; Loyd et al. 2013). Nonetheless, measures of prey returned provides a useful index of minimum prey killed (Woods et al. 2003), and as our main aim was to determine whether owners perceived their cats predatory behavior in the context of prey returns, this method provides an adequate measure to assesses this.

Prey-return rates (0.81–1.89 prey cat^−1^ month^−1^) were within the range found across studies globally (Churcher and Lawton 1987; Baker et al. 2005; van Heezik et al. 2010; Tschanz et al. 2011), and prey composition reflected predation patterns throughout the UK with mammals the most common prey item followed by birds (Woods et al. 2003; Baker et al. 2008). Our results provide further support to the wider evidence that age is a key predictor of predatory behavior, with prey-return rates decreasing as cats get older. Additionally, heterogeneity in local habitat appears to influence prey returns; cats residing closer to farmland kill and return more prey. Increased wildlife abundance surrounding agricultural landscapes (Baker and Harris 2007) may create more opportunities for prey encounters (Barratt 1998), although this edge-of-village effect may not translate to similar patterns in other habitats or urban areas (van Heezik et al. 2010). Unsurprisingly, cats that spent longer outside were also more likely to return prey. Thus, favorable circumstances both physically, spatially, and behaviorally appear to drive hunting opportunities and prey kills in the domestic cat. Prey-return studies, such as this, highlight key traits that correspond with high predatory rates. In light of this, targeted management could prove to be very important, that is, enforcing management in biodiversity hotspots and/or targeting awareness campaigns at owners of young cats.

Despite observational evidence that cats kill large numbers of native animals, we are still unable to infer the direct impact of cat predation on wildlife. Such studies would require detailed surveys of both prey and cat populations, and manipulating cat populations experimentally is logistically challenging, requiring cat exclusion zones. Further complications arise from uncertainty regarding sublethal impacts (Beckerman et al. 2007), quantifying unreturned prey items (Loyd et al. 2013) and whether cat predation compensates for natural wildlife mortality or has an additive effect (Baker et al. 2008). Despite a lack of definitive evidence that cats are significantly detrimental to biodiversity, there are suggestions that precautionary action should be taken (Lilith et al. 2006; Calver et al. 2011). Consultation with stakeholders is the logical and necessary intermediate step between ecological studies and the enforcement of mitigation strategies.

Deciding on potential management regimes is a complex problem with no single simple solution. The main stakeholders in this study, cat owners, are against control initiatives and do not accept that cats are harmful to the environment, a conclusion made irrespective of whether their cat is highly predatory. Although these opinions are from a limited area, the opinions are reflective of the general attitude of cat owners elsewhere in the UK (Thomas et al. 2012) and other countries where cat popularity is high (Ash and Adams 2003). Attitudes in the UK are dissimilar to those in Australia where the harmful effects of cat predation are widely accepted by the majority of cat owners (Lilith et al. 2006), and cat popularity is thought to be decreasing (Chaseling 2001). This could be a consequence of the wider publicity and enforcement of cat legislation in Australia and/or a greater awareness of the native wildlife emphasizing the negative implications of cat ownership. This tactic could be explored with UK cat owners although our simultaneous focus on the perceptions of owners and the predatory rates of their cats suggests simply telling owners the individual ecological impact of their cat is unlikely to alter their attitudes. In addition, although in Australia there appears to be a greater acceptance of the problem, Australian cat owners are still against total containment and cat exclusion zones (Grayson et al. 2002; Lilith et al. 2006), despite their enforcement in numerous areas (Denny and Dickman 2010). This implies that even the most informed cat owners may not be swayed by implications for biodiversity when the cost to the cat is perceived to be high. Instead, better motivation to accept controls on cat predation may be achieved by highlighting welfare advantages (Lilith et al. 2006; Toukhsati et al. 2012), such as lowering the risk of road traffic accidents, poisoning, infectious diseases, fighting-related injuries, and reduced threat from wildlife interactions, which are important contributors to cat mortality and long-term welfare (Moreau et al. 2003; Rochlitz 2004; Egenvall et al. 2010; Calver et al. 2013). In this study neutering, the only control strategy suggested not obviously linked to aiding wildlife populations and a largely welfare driven strategy was the most favored statement. We recommend further exploration of opinions of cat owners in the UK with a specific focus on the effectiveness of cat welfare as a motivational reason for owners to engage with controls on predatory behavior of domestic cats.

## Appendix

**Appendix A1:** Descriptions of predictive variables included in analysis of drivers of prey returns. Averages and ranges are included for continuous data types

Variable	Description
Distance from farmland (metres)	58.6 (9–180)
Age (years)	6.9 (0.75–17)
Estimated time spent outside per day (hours)	5.9 (1–14)
Sex (Male/Female)	24 F, 19 M
Wears a bell (Yes/No)	7 Y, 36 N
Food type (Dry/Both wet and dry)	13 D, 30 B
Inside at night (Yes/No)	14 Y, 29 N
